# Selections and Abstracts

**Published:** 1897-01

**Authors:** 


### SELECTIONS AND ABSTRACTS.

### The Modern Treatment of Diphtheria in Private Practice.

On the evening of September 14th, a little girl called at my office with a request that I should visit her sick brother. The public schools opened their doors on that day, and Johnny McD., although complaining of feeling sick, was sent to mingle with the hundreds of other school children.

An inspection of the case showed all the clinical symptoms of diphtheria, with a muco-purulent discharge from the nostrils so fetid that the odor filled the room. The examination completed, the mother anxiously inquired: "What is it?" "It is a case of diphtheria," I said; and her face blanched, her voice trembled as she said quietly: "I know what that means—I have buried two children with that."

On the 19th the father called at the office to say that Johnny could not be kept in bed, and they thought he was well.

In a skeleton way this illustrates the result of treatment with anti-diphtheritic serum, and stands in bold contrast with the drug treatment with the bottles of medicine, the cruel swab, the sleepless nights, the futile attempt to force food and medicine, the onset of secondary infection, and death or a tardy convalescence.

The uniform success which I have observed, and had in my own practice, has convinced me that the treatment of diphtheria with antitoxin is a great advance in therapeutics, and it is my impression that critics who have condemned this treatment have in most instances either observed only hospital patients, or have not persisted in the treatment, or perhaps have not had a fresh and reliable serum, or have not used it early enough.

From the standpoint of a general practitioner I confidently expect to cure any case of diphtheria in private practice seen within forty-eight hours of the onset of the disease.

Take, for instance, a typical case, a previously healthy child six years of age. The family physician is called in and finds the following conditions: general depression, face pale, pulse accelerated, temperature about 101° F. Inspection of throat shows general diffuse redness, with the characteristic deposit on one or both tonsils. This peculiar deposit once seen is not readily forgotten; the high fever, flushed face, and rapid pulse usually seen in pseudo-membranous tonsillitis are absent; the margin of the inflammatory process is usually sharply defined in diphtheria and not in tonsillitis. In follicular tonsillitis the leading symptoms are: intense congestion of the tonsils, with small discrete white patches, pulse and temperature high.

If, however, the symptoms are not well defined and the differential diagnosis cannot be clearly made, we should give the patient the benefit of the doubt and a dose of anti-diphtheritic serum administered at once. Then a culture should be made to verify the diagnosis. If I believe the case to be diphtheria, or have a reasonable doubt as to the diagnosis, I use the antitoxin whilst waiting for the report from the bacteriologist. If the case turns out to be tonsillitis, no harm has been done, as I consider a fresh, reliable serum, properly administered, devoid of danger.

Given, then, a case where the diagnosis of diphtheria is clear, I give as quickly as possible either 1000 units or 1500 units of the serum. The attendant is instructed to keep the throat clean with a bichloride solution of 1 to 5000; or a solution of permanganate of potash may be used, 1 to 400, if the attendant is not a trained nurse. With a young child difficult to manage, it is best to inject the solution into the nostrils; in older children a spray can be used in both the nostrils and throat more advantageously.

At the end of twenty-four hours I expect to find the membrane beginning to shrivel and curl up at the edges. In any event, however, I administer a second injection at this stage of the disease, and in a majority of instances this is sufficient. I advise very strongly that the second injection be given in all cases where the diagnosis of diphtheria is clear. I do not expect a cure from one injection, and rarely omit the second. If the symptoms do not indicate the beginning of convalescence at the end of forty-eight hours, I give a third injection. In fact, I would use a fourth injection if it seemed advisable at the end of another twenty-four hours, but I think this will rarely be found necessary.

I have not used anti-streptococcic serum, but I am convinced that in cases in which the treatment has been delayed, or in cases showing the streptococcic infection, proved by bacteriological investigation or from the peculiar red zone of inflammation which begins to spread from the margin of the diphtheritic process, the anti-streptococcic serum should promptly be used. Not only would I do this, but in cases of severe acute disease in the throat, which present all the symptoms of diphtheria, but where the bacteriological report does not confirm the diagnosis, I would resort to the anti-streptococcic serum. In fact, if I should have a case of diphtheria in which the membrane does not begin to peel up by the end of twenty-four hours, following, say the second injection of antitoxin, I will use the anti-streptococcic serum.

The importance of a fresh, reliable, highly concentrated serum must not be lost sight of; and as I have full confidence in our American product, I do not use imported serums. I have used several serums, but I have been best satisfied with the effects of that sent out from the biological department of Parke, Davis & Co. I heartily approve of the way this firm now puts up the serum in bulbs instead of bottles. It is not only highly concentrated, but, being hermetically sealed, should keep indefinitely. It is put up in bulbs of so many units, 250, 500, 1000, 1500; and each bulb being a dose, there is no temptation to use a serum that has been exposed to the atmosphere.

As to the medicinal treatment, I do not give any drug with the idea of influencing the course of the disease. I treat the conditions as they arise symptomatically. If I have evidence of the absorption of poisonous secretions, and a coated tongue, I give calomel tablet triturates, $\frac{1}{4}$ grain every hour, until the bowels move freely. Alcohol is rarely needed in cases receiving the serum treatment, especially if it is used early enough, whereas, under the old treatment, when we were so apt to find profound toxic symptoms, alcohol was more often needed. It is perhaps well to state here that I prefer fluid nourishment, principally milk, during the course of the disease.—W. A. WALKER, M.D., in *Pediatrics*.

---

### HYDROZONE IN GASTRIC AND INTESTINAL DISORDERS.

A period of nearly twelve years has elapsed since I first began the clinical use of hydrogen dioxide, generally referred to at that time as the peroxide of hydrogen. In 1887 I published a paper giving a detailed account of several cases in which it had been employed by inhalation, but even then I was thirty years behind the report of Dr. (now Sir) Benjamin Ward Richardson, of London, who had made a thorough investigation of its antiseptic, detergent, and healing properties. Notwithstanding the fact that this preparation had been known to the medical profession for that length of time it had achieved little or no reputation. This, however, may be explained by the fact that the discovery preceded the dawn of bacteriology. Indeed, I was one of the early contributors to medical literature relating to the clinical value of this product, and since that time I have published a number of articles, embracing practically every application, both medical and surgical, to which hydrogen dioxide is adapted.

In the present communication it is my object to direct the attention of the profession to its special value in the treatment of gastric and intestinal disorders. In gastritis, for example, there is no antiseptic which can be given with so much benefit as this remedy, because its effect is immediate, and even in considerable doses it is absolutely harmless. The same is true in regard to its employment in typhoid fever, cholera infantum, and Asiatic cholera. In the latter disease its efficacy has been thoroughly demonstrated by a number of well known physicians, and its applicability in cholera infantum is well known to those physicians who have given careful attention to the most modern methods in the treatment of this class of cases.

The following brief notes will be sufficient to indicate the availability of this remedy in the treatment of the disorders already mentioned, although, in view of the fact that hydrozone is a more concentrated product, and withal a permanent solution, this latter remedy should have the preference. It contains at least double the volume of nascent oxygen, which has heretofore been the standard for the medicinal peroxide of hydrogen.

In gastritis, either acute, subacute, or chronic, we have to deal with an unhealthy condition of the lining membrane of the stomach. The inflammation is attended with an increased output of mucus, which seriously interferes with the normal functions of the peptic glands. By the introduction of a small quantity of hydrozone, in the strength of one part to thirty-two parts of boiled or sterilized water, this objectionable mucus is at once destroyed by the action of the oxygen which is released, and the contents of the stomach remaining are promptly discharged into the small intestine. A patient suffering from gastritis should take at least half an hour before meals from two to four ounces of diluted hydrozone (one to thirty-two), and lie on the right side so as to facilitate the action of the stomach in discharging its contents.* The antiseptic properties of the hydrozone thus used are sufficient to destroy the micro-organisms and leave the stomach in a healthy condition for the absorption of nutritive pabulum. All forms of fermentation are promptly subdued by the active oxidation resulting from the liberation of the nascent oxygen. The patient is then in a condition to take suitable food, which should be nutritious and easily digested, liquids being preferred until the active symptoms have subsided. Later, small portions of solid food can be ingested, but all foods of a starchy character must be thoroughly masticated, in order to secure the action of the salivary secretion upon the starch granules, breaking them up, and lessening the tendency to fermentation in the stomach. After taking a meal, a patient with gastritis should follow it with medicinal doses of glycozone, which contain, in addition to the nascent oxygen contained in hydrozone, a percentage of glycerin which favors osmosis and assists in re-establishing the functional activity of both the peptic and mucous glands of the organ.

In the treatment of cholera infantum, typhoid fever, and Asiatic cholera, the same general plan should be adopted in dealing with the stomach, always bearing in mind the necessity for having the

* In chronic cases with a large amount of gastric mucus, and particularly in gastric ulcer, concentrated solutions are not well borne at first, owing to the formation of oxygen gas, but this difficulty disappears with the continued use of the remedy, and no treatment of gastric ulcer can be regarded as complete without the local employment of hydrozone.

patient remain in the recumbent position and on the right side for at least half an hour after the ingestion of the solution. In addition, however, to the preliminary treatment of the stomach, the same solution (one to thirty-two) is used as an injection into the lower bowel, care being exercised to insure its introduction as high up as possible. This can be managed by having the patient lie on the left side, with the hips well elevated, and the employment of a long, flexible rectal tube. In this manner we secure and maintain an antiseptic condition in both the stomach and large intestine, the importance of which will be understood when we consider the large number of micro-organisms which grow under these favorable conditions with such remarkable rapidity.

When deemed advisable, the solution introduced into the lower bowel may be combined with large quantities of either hot or cold water, which enables us to obtain the benefits of irrigation in addition to the antiseptic effects. These irrigations may be employed as frequently as deemed advisable by the medical attendant, but they will usually prove satisfactory if administered at intervals of four hours.

Although brief, it is believed this communication will prove serviceable to a large number of practitioners who have hitherto found serious difficulties in counteracting the mephitic influences of bacteria in this class of disorders, and the clinical virtues of the remedy being now so fully recognized, no one will hesitate to adopt the methods suggested, which may be conveniently carried out, in addition to the usual routine treatment.—JOHN AULDE, M.D., in the *New York Medical Journal*, August 15, 1896.

---

### THE TREATMENT OF QUINSY.

In any suggestions as to abortive measures, I recognize the fact that we do not usually see a quinsy patient until the disease has been progressing for twenty-four or more hours. To abort it at this stage is out of the question, in almost any case. We cannot claim for any medicinal remedy or combination a positive abortive effect in a case of quinsy.

In every case it is advisable to give a good mercurial cathartic followed by a saline. Unless we are reasonably positive of the presence of pus, when the case is first presented, I do not believe the use of the knife is advisable at that time. We must not provoke the inflammatory action by a too hasty attempt to relieve our patient by incision. We have all too often seen a veritable stabbing process performed in and about the tonsil, in the fruitless endeavor to locate the abcess. It appears to me this might usually be avoided by previously assuring ourselves with a careful differential diagnosis.

For many years the stereotyped line of prescribing had been quinine, opium, guaiacum, sodium salicylate, aconite and belladonna, either singly or in compound, as the opinion of the prescriber was guided by experience. I have no word of criticism for these. They were good, and doubtless afforded a vast relief in the aggregate. But as all things must change, and we tire, even of friends at times, so when improvements are made we must accept them as such. More recently salol has come into quite general favor.

From 1892, when I listened to Dr. Newcomb in his exhaustive paper on the treatment of tonsillitis medicinally, at the meeting of the American Medical Association in Detroit, until about one year and a half ago, I have felt that salol was the remedy *par excellence* for these cases. At that time three cases, all clerks in same department of one of our largest establishments, presented themselves at the same time. Two of them roomed together, the other lived with his parents. Such a coincidence at least suggested the possibility of contagion. A broken window on a cold, damp day was the presumable etiologic explanation. They were all suffering intense pain. Two of them had typical attacks of quinsy, the other tonsillitis. I used my usual applications, advised hot gargles and poultices, prescribing salol with rather confident promises of speedy relief. I had had two positive and two doubtful failures with salol in three years' experience previously, so that to find my patients growing worse for thirty-six hours did not entirely surprise me. Free incision was meanwhile made without obtaining pus, in either case of quinsy. The tonsillitis was further advanced, and was much relieved by the incision. In the other two I prescribed lactophenin, 10 grains every three hours; after the second dose Mr. B. was almost entirely relieved of pain; the temperature the following morning was 99.2°. The other symptoms were likewise very much ameliorated. In the case of Mr. R., the third dose relieved him quite as completely. At this visit I was able to thoroughly evacuate the pus and cleanse the cavity, affording the usual relief in such cases.

Since the above experience I have used the remedy in twelve cases of quinsy, and in all but one instance the results have been most gratifying to myself, and I am sure not less so to the patient. These patients have been first seen in all stages of the disease, from the first hour of the attack to the fourth day; and in one case, in consultation, on the sixth day.

The average time of relief has been about four hours. In all but three the relief was decided before the knife was used. In each of these three there were evidences of pus present, and the bistoury was used at once; so that the part played by the remedy is indeterminate.

I have in these cases given the lactophenin to the exclusion of every other remedy internally, excepting the cathartic already referred to; not omitting, however, the usual hot gargles and external applications.

My reasons for preferring it to salol are: Its action is decidedly more prompt; it has thus far given me no undesirable after-effects; it not only relives the pain but reduces the fever with an equal certainty. In cases of evident rheumatic diathesis I should certainly employ, in addition thereto, my customary remedies.—J. HOMER COULTER, M.D., in *Journal of the American Medical Association.*

---

### EXCISION AND SKIN-GRAFTING FOR TUBERCULAR DISEASES OF THE SKIN.

My experience is limited to two cases, and these are naturally insufficient for the formation of an opinion as to the merit of the method. Given a localized tubercular lesion of the skin, of not too great area, the pathologic condition would seem to justify the suggested treatment. Actual metastases, as in "cancer," are not to be expected, neither do we anticipate such an insidious creeping along the lymphatic channels. We do have a localized inflammatory condition, with tubercular structure and usually few bacilli.

The ordinary treatment is slow and generally painful. In lupus vulgaris, especially, the scar often presents a recurrence of the disease. If we can, under thorough antisepsis and asepsis, totally remove the disease, without infection of the wound with the tubercle bacilli, and then by grafting minimize the resultant scar, we have a method superior to the old.

Surgeons and some dermatologists recognize the value of excision, but I believe the procedure unpopular with the majority of dermatologists. Thorough excision, with care not to re-infect the wound surface, followed by proper grafting, should enable us to end the treatment of a not too extensive case within two weeks. Ordinary methods, especially in lupus vulgaris, often occupy years. White, in advocating excision, says that the bacilli may finally find their way to vital organs if the local disease persist too long. Here it might be asked if the general tubercular condition certainly has its origin from the local lesion, or are not all the tissues, in cases ending thus, in so favorable a condition for the bacilli that they receive them just as easily through other channels?

My two cases were treated at my clinic at the Atlanta Medical College. The first was that of a mulatto girl, aged 15, with a history of suppurating tubercular glands in the neck for a year, which finally healed, about a year before the appearance five years ago of the disease upon the lip. The lesion was upon the surface of the left segment of the lower lip, being about the size of a silver quarter, occupying nearly half the thickness of the lip. It was an ordinary lupus vulgaris, with fine nodules, covered with thin scales, save in the center, which was crusted and papular. There was a marked, firm, red, raised border.

The patient was anesthetized, and as strict asepsis as practicable was employed. The button "point" of a Paquelin cautery at a dull red heat was used to cauterize the tissues diseased and kill the bacilli. The entire patch was then carefully dissected away, allowing sufficient margin of healthy tissue, and to the depth of about one-half the thickness of the lip. Small grafts were then taken from the previously prepared thigh and properly applied. About 80 per cent. of these lived. A few more grafts were applied two weeks later to points left vacant by the slipping of some of the first. The success of the treatment was vitiated by some error in asepsis in later dressings. After some weeks a faint vesicular point was noticed at the inner edge of the healed surface, but this soon disappeared spontaneously. There was also a tendency to keloidal growth of the cicatrix, but secondary contraction soon relieved it.

My second case was that of a white boy of 12 years, in a poor general condition. Occupying the dorsum of the right wrist was an oblong, transverse, flabby, irregular ulcer, with an unhealthy base and soft, indolent, pale, undermined edges—in short, the lesion formerly described as a scrofuloderm. I believe the duration of the disease was over a year.

Under anesthesia and with asepsis, a free incision, well outside the margins of the lesion, ovally, without lifting the knife, was carried around the diseased tissues. A clean dissection was then made from the annular ligament, care being had not to allow the lesion, or anything which had been in contact with it, to touch the wound. Large grafts were shaven from the skin of the thigh and applied in the usual manner. The grafting was a failure, but a good, flexible scar was obtained. In addition to the usual dressings, a splint was used to restrain motion.

Inability to see or hear from my patients directly prevents my reporting their present condition. The first case seemed cured when seen two or three months after her discharge; the second is said to have had a "recurrence" in the scar.

My two cases simply demonstrate what I believe to be the proper method of operation, while they also constitute a warning as to the absolute necessity of thorough asepsis both at the operation and in the after-dressings. The second case also needed considerable preparatory treatment, which our haste to operate too often causes us to neglect. Excision without grafting is not so promising as the two combined, but perfect technique and extreme care in the later dressings would obviate much of the danger of future trouble in the cicatrix. We hope with grafting to get a minimum of granulation, and, later, scar tissue, which offers a minimum of liability to recurrence.

The cutaneous tuberculoses being now classed in one group, we can well generalize in their discussion, with a special allowance for the marked tendency of lupus vulgaris to return in its scar tissue.

Note.—The lupus patient was seen a few months after this paper was read. A young brother had died of tuberculosis meanwhile. The site of operation showed entire cure of the lupus. The cicatrix was still a little nodular and pigmented, though smoothing and whitening at edges. The fine hair included in grafts from leg had become long and coarse. She declined their removal by electrolysis. The final result in this case is satisfactory.

—Dr. M. B. Hutchins, in *Jour. Am. Med. Assn.*, Dec. 12, '96.

---

### Recent Progress in the Knowledge of Hereditary Syphilis.

It has not yet been demonstrated that syphilis is of bacterial origin, but our knowledge of this far-reaching infection has been greatly amplified and modified by clinical and pathological discoveries, which have been made while studying the nature and course of other infectious diseases. It is now definitely known that in many cases there is an intimate association of symbiosis of many morbid states and conditions, and that syphilis pathologically modifies other morbid conditions. In its turn, its pathological developments may in like manner be acted upon by other diseases. These facts have been well presented in a short essay on hereditary syphilis and tuberculosis by Hochsinger, which will undoubtedly lead, in time, to a revision and recasting of all our knowledge of the pathological anatomy of inherited syphilis. He gives the history of three cases in which the symbiosis of syphilis and tuberculosis was positively proved, and these histories quite clearly show that the latter infection was the predominating one. Had not these cases been so thoroughly studied, both histologically and bacteriologically, they would, like many thousands before them, have been incontinently set down as typical examples of heredity with visceral lesions.

As a result of these studies and observations, Hochsinger concludes that a mixed infection of hereditary syphilis and tuberculosis may be found in the very youngest children, and that thus a double infection may be simultaneously transmitted at conception. These observations also show that all nodules, caseous or otherwise, found in hereditary syphilitic infants should not be pronounced to be syphilitic gummata until it is shown that they do not contain the tubercular bacillus. In hereditary syphilitic pneumonia there is true interstitial granulation-tissue infiltration, with perivascular cell-proliferation into the lung-parenchyma, and no tubercular bacilli.

The foregoing considerations very clearly emphasize the facts that in many bad cases of hereditary syphilis there is, in all probability, a synchronous tubercular infection, and that no nodules found in the viscera, and, indeed, we may add, in the bones, joints, and skin of these subjects, should be pronounced to be gummata until the influence of tuberculosis has been excluded. Diagnoses which are based on microscopical appearances are not reliable, since to the eyes the lesions of syphilis very often resemble those of tuberculosis in a striking manner. In some nodules there has been found evidence of the action of both syphilis and tuberculosis.

In earlier days we were accustomed to speak of the cause of death of infants and children hereditarily syphilitic as due to congenital want of vitality, particularly when, at the autopsy, we found no visible involvement of an important organ. Pavloff (*Annals de Dermat. et de Syphiligraph*, July, 1895) concludes that vessel-changes are the cause of death of most hereditarily syphilitic children, and he makes the pertinent suggestion that this extensively distributed morbid condition indicates the great necessity of a general systematic treatment. It is important, in this connection, to remember that Mracek found in the viscera of eighteen hereditarily syphilitic children hemorrhages of such severity that they constituted the active cause of death. As bearing upon Pavloff's conclusions, Bar and Tissier, at the autopsy of a child at term, whose parents were the victims of active syphilis, found pathological changes in the veins and arteries throughout the entire body.

The histories of cases reported by Murri, Schumacher, and Gœtze have shown that there is a probable etiological relation between acquired syphilis in some subjects and paroxysmal hemoglobinuria. Recent observations seem to show that the morbid combination may occur in the course of hereditary syphilis. Courtois-Suffit (*Médecine Moderne,* March 2, 1895) reports the case of a four-year old child born of syphilitic parents, presenting characteristic lesions of the tibia, who, during two years (third and fourth of life), suffered from attacks of hemoglobinuria, at first when exposed to severe cold, and later on even when the temperature was not low. Treatment by mercurial inunctions and the ingestion of potassium iodid caused improvement in the osseous lesions and allowed the child to escape any attacks of hemoglobinuria during a severe winter. Comby, in a later communication (*Ibid.,* March 6, 1895), claims that he had previously pointed out this symptom as being etiologically related to hereditary syphilis. Gœtze reported the case of a child, nine years old, who presented structural evidences of hereditary syphilis, but whose medical history was unknown, who suffered from hemoglobinuria. The study of this interesting question has only just begun, it being yet in the stage of case-reports.

A like condition prevails as to the clinical history and the pathology of the changes and disorders of the kidneys in hereditary syphilis. Hoch details the case of a typical syphilitic infant, three months old, in which, while favoring the view that the nephritis was caused by syphilis, he suggests that perhaps it was due to the action of mercurials. In this case the hiatus consists in the absence of an autopsy and of histological findings.

In a case reported by Massalongo, we find more satisfactory data (*Arch. per Scienze Mediche,* lx. and ii., 1895); the infant was six months old, born of a syphilitic mother, and it presented typical lesions. Before death it suffered from albuminuria, edema, convulsions, and uremic coma. Histological examination of the kidneys showed well-marked changes in the arteries—endarteritis and periarteritis—and thromboses, extensive and interstitial nephritis, and great increase in the connective tissue, which compressed the glomeruli and produced schlerosis of the organ. The lesions resembled those of late nephritis in acquired syphilis of adults. Massalongo thinks that these visceral changes began in intra-uterine life.

With the expansion of our knowledge of syphilis as a chronic infectious disease, it seems that many surprises are in store for us. Cases reported by Hutchinson, D'Ornellas, Morgan, Elsenberg, and myself, have shown very clearly that Raynaud's and Morvan's diseases may occur in the course of acquired syphilis, and that the latter infectious process is, in all probability, etiologically related to them. Krisowski (*Jahrbuch f. Kinderheilkunde*, vol. xi., pp. 57 *et seq.*) described a well-observed case of hereditary syphilis, in which symmetrical gangrene was a prominent symptom. Under mercurial inunctions and the internal use of potassium iodid a cure was promptly brought about. Here, then, is another subject for study in the protean drama of hereditary syphilis.—TAYLOR, *Medical News.*—*Polyclinic.*

---

### TREATMENT OF SYPHILODERMATA.

A careful consideration and trial of the various methods of treating the syphilodermata has led me to the following conclusion :

1. In the primary stage, when only the chancre is present, no general treatment; calomel locally.

2. As soon as the secondary period sets in, as shown by the general adenopathy, angina, cephalalgia, and eruption, the internal treatment for mild cases should be $\frac{1}{4}$ to $\frac{3}{4}$ of a grain of the protoiodide of mercury t. d., continued for three months, or until the symptoms disappear. In severer cases, with pustular eruptions, severe anginas, persistent headaches, etc., a course of 6 to 10 intramuscular injections of 10 per cent. calomel-albolene suspension, 5 to 10 minims at intervals of five to fifteen days, should be employed.

3. After completion of the course and cessation of the symptoms, employ tonics, etc., without specific treatment, for three months.

4. Thereupon a second calomel course as above, plus a small dose (15 grains) of iodide of potassium in milk after meals. This to be given whether later secondary symptoms of the skin and mucosæ appear or not.

5. Second intermission of treatment, lasting three to six months, according to the presence or absence of symptoms.

6. In the second year, if tertiary lesions marked by deeper and more localized ulceration are present, give the iodide of potassium in increasing doses (60 to 600 grains daily) as may be necessary. Combine with it occasional courses of calomel injections. If no lesions appear, give a mild course of both.

The best local treatment of the syphilodermata is with the mercurial plaster-mull.—Dr. W. S. GOTTHEIL, abstract of lecture at New York School of Clinical Medicine.

---

### A GARGLE FOR FOLLICULAR AMYGDALITIS.

Levy (*Semaine medicale,* September 23, 1896; *Weiner klinische Rundschau,* October 11, 1896) recommends the following:

| | | |
|---|---|---|
| ℞ | Creosote | 8 drops. |
| | Tincture of myrrh, Glycerin, each | 900 grains. |
| | Distilled water | 1,800 grains. |
| M. | | |

—*N. Y. Med. Journal.*

---

### THE APPENDIX AND GRAPE SEEDS.

Dr. Edmund Andrews, of Chicago, believes (*Jour. Am. Med. Association*) that:

1. The appendix is not a "functionless" organ. It produces every day a quantity of tenacious mucus to lubricate the cecum, and by thus facilitating the fecal movement prevents impaction in the head of the colon.

2. The current of this tough mucus is toward the gut, hence seeds and other foreign bodies cannot enter the appendix in opposition to the movement as long as the organ is in a healthy condition.

3. From various causes perforations may occur in the appendix. The current of mucus is then reversed and flows outward, and small bodies in the colon may thus be drawn into the appendix, or even carried through it into the abscess or the peritoneum without being the cause of the perforation.

4. There is no scientific proof that grape seeds are any more dangerous than the hundreds of other small objects which we daily swallow with our food.

---

### THE TREATMENT OF ACUTE OTITIS MEDIA AND SUPPURATIVE MASTOIDITIS.

Hessler states (*Am. Jour. Med. Sci.*) that in acute otitis media with imperforate membrane he makes a large curvilinear incision in the membrane, extending from its anterior border beneath the malleus to the middle of its posterior periphery. In all acute cases he strictly forbids syringing, but advises a patient to mop the ear gently with cotton wool as often as is required. Cotton wool should not, however, be worn in the ear. Inflation of the tympana in the acute stages is strictly forbidden, as this procedure may produce further inflammation by infection from the nasopharynx. Should secondary mastoid inflammation supervene, operation by Schwartze's method is recommended. The resulting wound should not be irrigated, but simply tamponned with iodoform gauze.

In the discussion which followed the reading of this paper, Panse (Dresden) remarked that patients should not be allowed to mop their own ears with cotton wool. He places strips of iodoform gauze in the auditory canal in cases of acute discharge, allowing the secretion to run into a bandage, which is changed as often as is necessary.

Stacke (Erfurt) stated that in acute suppurations he avoided not only syringing, but did not allow the patient to assist in any way in the treatment.

Leutert (Halle) advised no syringing, but that the external meatus be kept closed, in order to prevent entrance of air.

Brieger (Breslau) favored dry cleansing with sterilized cotton wool.

Hartman (Berlin) said that he did not employ inflation in acute cases, nor irrigation of the wound after mastoid chiselling.

Reinhard (Duisburg) also spoke against irrigation in acute cases.

Walb (Bonn) avoids inflation or the use of the catheter for fear of forcing pus into parts previously unaffected.—*Med. Chronicle.*

### THE ABUSE OF THE STIGMATA OF DEGENERACY.

In that late entertaining book, "Over the Hookah," by Dr. Frank Lydston, of Chicago, the "talkative doctor" sounds an important word of warning respecting the use, or rather abuse, of the stigmata of degeneracy in certain expert testimony:

"The modern school of criminal anthropology has accomplished much, but it does not claim to have established as yet, any data that warrant our making the stigmata of degeneracy an important issue in murder trials. But dabblers and pretenders infest every art and science, and we must console ourselves with the reflection that such persons by no means represent scientific thought. . . . The worst feature of such notoriety-seeking fellows is that they drag 'star-eyed science' in the mire and make it an object of ridicule and distrust. The so-called science of phrenology, absurd as it is, was never half so ridiculous as the sham science that certain camp-followers in the field of criminal anthropology and the study of abnormal man are endeavoring to set up. . . . Modern science holds, not that certain peculiarities of development necessarily indicate a particular type, or indeed, any type, of insanity or criminality, but that the criminal and the insane are characterized on the average by aberrations of cranial development and certain other departures from the normal average standard, that we term stigmata of degeneracy—a wide distinction, and a still wider difference. To be sure, there are extreme types of atypical cranial development that are associated with certain peculiar mental attributes, but not with sufficient frequency to form a basis for dogmatic expert evidence. . . . I do not deny that there is something suggestive in cranial conformation—we hope there may yet be more—but it is best to be very conservative in our judgment on this point. Lombroso and his school have taught us much, but let us not claim more than our masters. Science sometimes has need to cry 'Save me from my friends.'"

---

### HOW TO WRITE A MEDICAL PAPER.

Dr. W. P. Northrup, of New York, has made the following suggestions as to medical writing, with which all editors will agree heartily:

Scratch out your introduction and begin where the subject really begins. Condense the body of the paper. End the paper where the subject ends. Successful papers, almost without exception, are those written with one definite and predominating thought, on which every fact is brought to bear and toward which every argument is directed. Conclusions alone are, as a rule, sufficient, with pertinent facts so marshaled as to give them proper support. The various minute details of the stages by which these conclusions are reached are usually uninteresting, and had better be touched on lightly or omitted entirely. An expert editor, by remorselessly stripping away the padding, is usually able to make an abstract that will present all the author's ideas and conclusions in one-fourth the space of the original paper.

Many a man who has had something of real value to say has first smothered the life out of it with padding, and then dug a grave and buried it in the midst of a five-column paper compiled from some text-book. It would be far better for professional literature if every man would content himself with writing what he really knows, instead of writing what he has only read. One new fact discovered, one new, live, practical idea, is a sufficient subject for one paper, though it may be a short one. Two or three subjects for a single paper will render it weak or actually inert. A shot-gun is adapted to small game, but large game is only brought down with a rifle. A single paper on a live subject, if it hits the mark squarely, will do more to establish a man's reputation than ten diluted and watery compilations.

---

### Some Good Expectorants and Cough Remedies.

The following formulæ have been thoroughly tested by Dr. James K. Crook, of New York, in hospital and private practice, and may be trusted to render good service in properly selected cases:

1. For irritative coughs: ℞ Phenacetin, 20 to 40 grains; ext. glycyrrhizæ, 20 grains; sacch. albi, 2 drachms. Fiat pulvis, in chartulas xx. dividendus. Sig.: One to be taken at one, two, or three hour intervals.

2. When an expectorant effect is desired: ℞ Extracti glycyrrhizæ, 20 grains; phenacetin, 20 to 40 grains; ammonii muriatis, 1 to 2 drachms; sacch. albi, 2 drachms. M. et in chart. xx. div. Sig.: One powder to be taken in a little water every two, three, or four hours.

3. A good stimulating expectorant for adults: ℞ Apomorph. hydrochloratis, 1 grain; syr. ipecacuanhæ, 2 fluidrachms; syr. tolutani, 1 fluidounce; aquæ dest., q. s. ad. 3 fluidounces. Ft. mist. A teaspoonful five times daily at four-hour intervals. Shake well before using. Prepare freshly as required and keep in a dark-colored bottle.

4. A good stimulating expectorant for every-day bronchial and phthisical coughs: ℞ Ammonii muriatis, 2 drachms; tincturæ opii camphoratæ, spiritus chloroformi, syr. ipecacuanhæ, āā 2 fluidrachms; syr. prun. Virginianæ, q. s. ad. 3 fluidounces. M. Sig.: A teaspoonful every three or four hours. Shake well before using.

5. For weak and fruitless coughs with loss of bronchial power: ℞ Ammon. carbonatis, 1 to 2 drachms; tinct. tolutani, 2 fluidrachms; syr. senegæ, spiritus vini gallici, syr. simplicis, āā 4 fluidrachms; aquæ dest., q. s. ad. 3 fluidounces. Ft. mist. Sig.: A teaspoonful in a little water every two, three, or four hours.

6. For asthmatic and emphysematous coughs: ℞ Spiritus ætheris compositi, 4 fluidrachms; potassii iodidi, ammon. muriatis, āā 2 fluidrachms; codeinæ sulphatis, 2 grains; syr. tolutani, 4 fluidrachms; aquæ dest., q. s. ad. 3 fluidounces. M. Sig.: A teaspoonful every two, three, or four hours.

7. For recurring bronchitis or winter cough: ℞ Terebene, 6 fluidrachms; ol. eucalypt., 2 fluidrachms. M. Sig.: Ten to fifteen drops on a little sugar every three or four hours.—*Medical Record.*

---

### EFFECT OF THE X RAYS UPON THE SKIN.

The editor of the *Journal of the American Medical Association* mentions this matter in his issue of December 12, 1896. He refers to a case reported by Dr. King (*Canadian Practitioner*, November, 1896) of an operator and exhibitor who was exposed to the rays for many hours a day for some weeks. Dermatitis, "other alterations in the skin," destruction of hair and nails—and conjunctivitis occurred. This case, as also Dr. Skinner's (*Jour. Am. Med. Assn.*, November 14), presented some of the symptoms of sun-burn, but other symptoms were rather peculiar.

Of course, as the editor says, these effects would not follow ordinary (necessary) and moderate exposure. The effect upon show people does not interfere with the scientific use of the rays. H.

---

### TREATMENT OF LUPUS VULGARIS BY MEANS OF ELECTROLYSIS.

Dr. A. Ravogli, of Cincinnati, read a paper upon this subject at the last meeting of the American Medical Association (published in *Jour. Am. Med. Assn.* Dec. 5, '96). He reviews the various methods of treatment, and mentions those who have used somewhat the same method as followed by himself. In the case reported he used a needle attached to the negative pole, and employed a current-strength of ten to twelve milliamperes. The needle was inserted deeply in the lupus nodules and the current allowed to pass for a minute or longer. He says the entire nodule is thus destroyed. He obtained perfect success in five weeks in his case, which involved the nose, lips, and cheeks.

Dr. Ravogli's method promises some advantages over the caustic and other plans of treatment, though—as was objected at the time his paper was read—ten to twelve m. a. produces an actual caustic effect, or one indistinguishable from a caustic effect. H.

---

### UNGUENTS FOR INSECT BITES.

Brocq suggests the following preparations for the bites of fleas, gnats, and bugs:

1. Camphorated chamomile oil, 100 grams; balsam of storax, pur. 20; essence of peppermint, 5 gr.

2. Olive oil, 20 gr.; ointment of storax, 25 gr.; balsam of Peru, 5 gr.

3. Naphthol, 5 to 10 grams; ether, q. s. to dis.; menthol 0.25 c. to 1 gram.; vaselin 100 grams.—*Jour. de Méd. de Paris*, October 18.—*Jour. Am. Med. Assn.*

